# P-glycoprotein-9-mediated multidrug tolerance in *Caenorhabditis elegans*

**DOI:** 10.1186/s13071-025-07091-5

**Published:** 2025-10-30

**Authors:** Clara Blancfuney, Eva Guchen, Anne Lespine, Mélanie Alberich

**Affiliations:** INTHERES, Université de Toulouse, INRAE, ENVT, Toulouse, France

**Keywords:** Anthelmintic, Macrocyclic lactones, Resistance, *Caenorhabditis elegans*, P-glycoproteins, Amphids, Nuclear hormone receptor, NHR-8, PGP-9

## Abstract

**Background:**

The active drug efflux pumps P-glycoproteins (PGPs) are the cornerstones of multidrug resistance in many organisms. In parasitic helminths, resistance to macrocyclic lactones (MLs) has been associated with *pgp* regulation and structural defects in amphids. In *Caenorhabditis elegans*, the nuclear hormone receptor (NHR)-8 also influences xenobiotic tolerance by regulating *pgp* genes. However, the specific contribution of individual transporters and their regulation remain poorly defined. We recently demonstrated that PGP-9 specifically contributes to ivermectin (IVM) tolerance in an IVM-resistant *C. elegans* strain. This study aimed to explore the role of PGP-9 in drug efflux in *C. elegans*.

**Methods:**

We used the IVM-resistant and dye-filling defective (Dyf) *C. elegans* strain IVR10 and a *pgp-9* IVR10 mutant to assess larval development under MLs (eprinomectin (EPR) and moxidectin (MOX)) and tunicamycin (TM). We evaluated whether the Dyf phenotype was affected by *pgp-9* deletion. We investigated the role of NHR-8 in regulating *pgp-9* via reverse-transcription quantitative polymerase chain reaction (RT-qPCR) and by assessing ML sensitivity in an IVR10 *nhr-8* mutant. Additional candidate regulators of *pgp-9* were also tested.

**Results:**

IVR10 displayed resistance to MLs and to TM, while *pgp-9* deletion restored full drug sensitivity despite the persistence of the Dyf phenotype. Although *nhr-8* deletion in IVR10 increased IVM sensitivity, *pgp-9* expression was not significantly altered in that strain or IVR10. Interfering RNA (RNAi) targeting *pgp-9* in the *nhr-8* mutant further increased IVM sensitivity, uncoupling PGP-9 from NHR-8 regulation. Candidate NHRs did not affect IVM tolerance in N2B.

**Conclusions:**

These results provide the first direct evidence that PGP-9 is necessary for multidrug tolerance in *C. elegans*, independently of amphid structural defects and NHR-8 regulation. These findings uncover a novel mechanism supporting drug resistance and highlight PGP-9 as a potential therapeutic target to improve ML treatments.

**Graphical Abstract:**

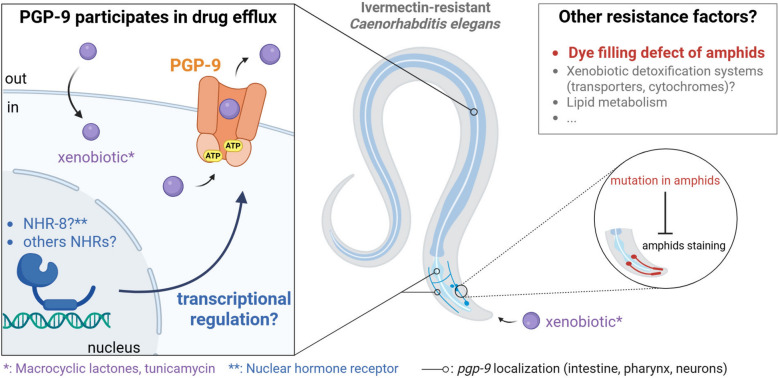

## Background

P-glycoproteins are ATP-binding cassette (ABC) efflux pumps of the plasma membrane. Owing to their ability to transport a wide variety of substrates, they stand as one of the major contributors to multidrug resistance in various organisms [1–3]. They are notably suspected of participating in tolerance to macrocyclic lactones (MLs) [3], which are broad-spectrum anthelmintics (AHs) and the cornerstone of gastrointestinal helminth control (e.g., *Haemonchus contortus* and *Teladorsagia circumcincta*) in the livestock industry [4]. Among them, ivermectin (IVM) is one of the most important human and veterinary drugs available today [5], and was awarded the Nobel Prize in Physiology and Medicine in 2015 for its remarkable antiparasitic properties and contributions to global health. Moxidectin (MOX) [6] and eprinomectin (EPR) are two other MLs of importance. Unlike IVM, both have minimal or no milk withdrawal period [7], which is a significant advantage in dairy farm production. The intensive use of these drugs has inevitably led to the emergence of drug-resistant parasite populations [8], which often exhibit cross-resistance to other compounds of the same drug class [9]. Because MLs are the primary line of defense against parasites, understanding the mechanisms underlying resistance and the contribution of PGPs stands as an urgent challenge.

To investigate these resistance mechanisms, *Caenorhabditis elegans* is a pivotal nematode model that offers several advantages over parasitic species, including a short life cycle, ease of maintenance, no host dependency, and the possibility of genetic modifications. More importantly, it is susceptible to MLs. Thus, it serves as a valuable tool to study ML resistance and was notably key in elucidating the mode of action of avermectins [10] or identifying potential actors of drug resistance [11]. Additionally, an IVM-resistant *C. elegans* strain (IVR10) was generated by stepwise exposure [12] and has since contributed in advancing our understanding of resistance mechanisms, notably by transcriptional studies, for example [13, 14].

Although several studies have demonstrated an association between PGPs and ML resistance, functional evidence for specific roles of individual PGPs in vivo remains scarce and fragmented across nematode species. Parasite PGPs have been shown to interact with MLs [15–17] and are overexpressed both in resistant field isolates [18, 19] and in the IVM-resistant *C. elegans* strain IVR10 [14]. However, their plurality and diversity across nematode species make it hard to describe their specific and individual role in resistance. For instance, the *C. elegans* genome harbors 15 *pgp* genes (including one pseudo-gene). These genes share relatively high sequence similarity [20] and, in some cases, originate from tandem duplications [21] or share overlapping expression patterns [22], making it difficult to assign a specific function to each transporter. Recently, our work revealed that PGP-9 [23] strongly potentiates IVM tolerance in IVM-resistant *C. elegans* IVR10 strains. We demonstrated that *pgp-9* knockout in IVR10 significantly increased IVM sensitivity compared with deletion of other *pgp*s. We showed that PGP-9 is expressed in the pharynx, the intestine, and in head neurons, an interesting observation given that glutamate-gated chloride channels (GluCls), which are the primary target of MLs, are also localized in neurons [24, 25]. We demonstrated that PGP-9 is essential to maintain IVM resistance by facilitating the efflux of an IVM fluorescent probe at key tissues [23].

The transcriptional regulation of *pgps* in *C. elegans* appears to be governed by the nuclear hormone receptor NHR-8 [11, 26], also known to regulate cholesterol and bile acid homeostasis [27]. Consistently, knockout or knockdown of *nhr-8* potentiates IVM sensitivity in *C. elegans* and *H. contortus*, respectively [11].

Besides PGPs, ML resistance is associated with a dye absorption defect in resistant nematodes [11, 24, 28, 29]. The dye-filling phenotype reflects the worm’s ability to internalize a lipophilic dye (Dil) that labels amphids, key chemosensory neurons of the worm. However, its functional significance and its specific contribution to resistance remain unclear. Interestingly, in *C. elegans*, resistance to tunicamycin (TM), which causes major endoplasmic reticulum stress and significantly reduces lifespan [30], has also been jointly associated with PGP detoxification and the dye-filling defect (Dyf) phenotype. In particular, in *osm-3* mutants, TM resistance seems to be mediated by PGPs, which are themselves regulated by NHR-8 [26].

Here, we aim to further investigate PGP-9-mediated multidrug tolerance mechanisms using the *C. elegans* IVR10 strain deleted for *pgp-9* (IVR10(Δ*pgp-9*)). Given the known cross-resistance profile of the parental IVR10 strain [14], we assess how the deletion of *pgp-9* affects sensitivity to other MLs (MOX and EPR) and TM. In parallel, we explore the transcriptional regulation of *pgp-9*, focusing on the role of NHR-8 [11] and the possible involvement of additional regulatory factors. Finally, we investigate how *pgp-9* deletion might impact the Dyf phenotype of the parental strain IVR10, which is associated with xenobiotic resistance [26, 31]. This study advances our understanding of PGP-9’s multidrug transporter functions and of the multiple, potentially interconnected, factors involved in ML resistance.

## Methods

### Materials

All chemicals were obtained from Sigma-Aldrich, unless otherwise stated. Ivermectin, moxidectin, eprinomectin, tunicamycin and 1,1′-didodecyl-3,3,3′,3′-tetramethylindocarbocyanine perchlorate (DiIC_12_(3)) were dissolved in dimethyl sulfoxide (DMSO), and the maximal concentration of DMSO was 0.5% in all assays.

### *Caenorhabditis elegans* strains and cultivation conditions

Wild-type *C. elegans* strain N2 Bristol (N2B), VC1298(*nhr-115*(gk579) V.), and the OP50 *Escherichia coli* strains were provided by the Caenorhabditis Genetics Center (CGC, University of Minnesota, Minnesota, Minneapolis, MN, USA). The *C. elegans* strains invalidated for *nhr-226* and *nhr-210*, i.e., tm1804 and tm1290, respectively, were obtained from the National Bioresource Project (Tokyo, Japan). The IVM-resistant strain IVR10 was kindly provided by C. E. James [12]. IVR10 strains deleted individually for *pgp-9* and *nhr-8*, i.e., IVR10; *pgp-9* (knu1140 [Δ9179bp]) [23] and IVR10; *nhr-8* (knu1123 [3320 bp]) were designed by InVivo Biosystems (Eugene, Oregon, USA). Full-length deletions were realized in the IVR10 genome using the clustered regularly interspaced short palindromic repeats (CRISPR)-sdm transgenesis method, and a three-frame stop sequence was inserted. In this study, these strains are referred to as IVR10(*Δpgp-9*) and IVR10(Δ*nhr-8*). The genotype of each strain was confirmed by PCR and sequencing.

Strains were cultured and handled according to the procedures previously described [11, 14]. Nematodes were cultured at 21 °C on Nematode Growth Medium (NGM) agar plates (1.7% bacto agar, 0.2% bacto peptone, 50 mM NaCl, 5 mg/L cholesterol, 1 mM CaCl_2_, 1 mM MgSO_4_, and 25 mM KPO_4_ buffer) seeded with *E. coli* strain OP50 as a food source. All *C. elegans* strains were cultured on classic NGM agar plates, except for IVR10, which was cultured on NGM plates containing 10 ng/ml of IVM.

### Synchronization

Nematodes were synchronized through egg preparation with sodium hypochlorite. An asynchronous population was collected from NGM plates with M9 buffer (3 g KH_2_PO_4_, 6 g Na_2_HPO_4_, 5 g NaCl, 0.25 g MgSO_4_·7H_2_O in 1 l of water). All larval stages except eggs were lysed with bleaching mix (5 M NaOH, 1% sodium hypochlorite) followed by M9 washes. *C. elegans* eggs were then hatched overnight with mild agitation at 21 °C in M9 to obtain a synchronized population of first-stage larvae (L1).

### Larval development assays (LDAs)

This assay measures the potency of a drug in inhibiting the development of *C. elegans* from L1 to the young adult stage. The LDA was essentially conducted as described previously [11, 14, 32].

#### Solid LDA

Thirty synchronized L1s were added per well of a 12-well plate. These were seeded on NGM containing increasing concentrations of a drug, supplemented  with OP50 bacteria. Each concentration was set up in triplicate. Plates were incubated at 21 °C until L1s of the negative control had developed into young adult worms. Development was calculated as a percentage of young adults in the presence of the drug, normalized to the untreated control. All experiments were reproduced in at least three biological replicates unless otherwise stated. Curve fitting for the LDA (sigmoidal dose–response curve with variable slope) was performed with the GraphPad Prism 8 software. IC_50_ values, the concentrations at which 50% of the animals fail to reach the young adult stage, and the resistant factor (RF), the fold resistance relative to N2B, were determined.

#### RNA interference on *pgp-9* in IVR10(Δ*nhr-8*)

RNA interference (RNAi) was conducted by feeding HT115 bacteria transformed with L4440 vector that produces double-stranded RNA against a targeted gene in the strains. HT115 bacteria clones expressing *pgp-9* RNAi or the empty vector as control from the Ahringer RNAi library were grown for 8 h at 37 °C in LB medium containing ampicillin (50 μg/ml). They were then seeded on NGM plates supplemented with carbenicillin (25 μg/ml) and isopropyl β-d-1-thiogalactopyranoside (IPTG, 1 mM) to induce RNAi expression. Finally, solid LDA was conducted as described above. The efficiency of knockdown of *pgp-9* mRNA was verified in a previous study [23].

#### Liquid LDA

This assay was performed in liquid media to rapidly screen for *nhr* genes. In that case, 25 synchronized L1s were seeded in 200 µl of complete liquid S-Basal media supplemented with OP50 (5 mg/ml). Complete medium was prepared as follows: 50 ml of S-Basal (5.85 g NaCl, 1 g K_2_HPO_4_, 6 g KH_2_PO_4_ in 1 l of water), 500 µl of potassium citrate 1 M pH 6 (20 g C₆H₈O₇, H₂O, 293.5 g K_3_C_6_H_5_O_7_, H_2_O in 1 l of water), 500 µl of trace metal solution (1.86 g C₁₀H₁₄N₂Na₂O₈, 0.69 g FeSO_4_·7H_2_O, 0.2 g MnCl_2_·4H_2_O, 0.29 g ZnSO_4_·7H_2_O, 0.025g CuSO_4_·5H_2_O), 150 µl CaCl_2_ 1 mM, 150 µl MgSO_4_ 1 mM, 50 µl cholesterol 5 mg/ml. Drug treatment was administered by adding 1 µl of IVM at increasing concentrations. Plates were incubated at 21 °C under gentle agitation until L1s of the negative control had developed into young adult worms. Development, dose–response curves, IC_50_s, and RFs were determined as described above.

### Dye-filling assay of amphid neurons

Amphids were dyed according to a previously described procedure [14]. *C. elegans* strains N2B, IVR10, and IVR10(Δ*pgp-9*) were synchronized and then incubated at the L4 stage in DiIC_12_(3) (1,1′-didodecyl-3,3,3′,3′-tetramethylindocarbocyanine perchlorate) in M9 at 10 ng/ml under gentle agitation for 2 h at 21 °C. Worms were then washed in M9 and allowed to recover on NGM plates for 3 h. After that period, they were paralyzed with 2.5 mM sodium azide and imaged on a SMZ800N fluorescent stereomicroscope (Nikon) coupled to an Intensilight C-HGFI (Nikon) with a red fluorescent protein (RFP) filter and visualized using a VisiCam 5 Plus (VWR). Animals with visible amphid staining were counted and normalized to the total number of worms.

### RT-qPCR

A population of 1500 synchronized L4 larvae was collected from NGM plates with M9 buffer in four replicates and flash-frozen in liquid nitrogen in RLT buffer (Qiagen) supplemented with dithiothreitol (DTT) 4 mM. Lysed worms were then stored at −80 °C. Frozen samples were thawed and homogenized three times for 10 s at 6 m s^−1^ in a FastPrep-24 instrument (MP-Biomedicals, NY, USA). Total RNA was extracted using RNeasy Plus Kit (Qiagen, S.A., Courtaboeuf, France) according to the manufacturer’s instructions. Total RNA was quantified using a NanoDrop ND-1000 spectrophotometer (NanoDrop Technologies Inc., Wilmington, DE, USA). Complementary DNA (cDNA) was synthesized from 1 μg of total RNA using a Maxima H Minus First Strand cDNA Synthesis Kit (Thermofisher).

Real-time quantitative polymerase chain reaction (RT-qPCR) was performed using SYBR Green PCR Master Mix (Applied Biosystems Life Technologies, Courtaboeuf, France) on QuantStudio 5 Real-Time PCR System (Applied Biosystems) at GeT-TRiX (ToxAlim UMR1331 INRAE/INP/UPS, Toulouse, France). The primers that were used are listed in Table 1. Results are expressed according to the relative quantification method with *tba-1* as the reference gene.

**Table 1:** Specific primers for *Caenorhabditis elegans* genes targeted by RT-qPCR

Gene	Forward	Reverse
*Cel-pgp-9*	CAAATCTGCTATTGATTATCGGCTC	CCGAGACCTCCACCAACTGA
*Cel-tba-1*	ATCGATTTTTGTAGATCTTGAGCCA	TCCAGTGCGGATCTCATCAAC

### Statistical analysis

All experiments were conducted independently at least in triplicate unless otherwise stated. Results are expressed as mean ± standard deviation (SD). Statistical analyses were performed using GraphPad Prism 8.4.2 software, and results were considered statistically significant when *P* < 0.05.

Pairwise comparisons in Tables 2, 4, and 5 were statistically evaluated with unpaired parametric *t*-tests or unpaired *t*-test with Welch’s correction, depending on the case. To evaluate the impact of *nhr-8* deletion in the IVR10 background compared with the control strains N2B and IVR10, a one-way analysis of variance (ANOVA) followed by a Tukey post hoc multiple-comparisons test was performed. The same test was conducted to assess the differences in Dil staining in N2B, IVR10, and IVR10(Δ*pgp-9*) and to compare *pgp-9* mRNA transcript levels between each strain.

**Table 2:** Comparative sensitivities of *Caenorhabditis elegans* strains to moxidectin (MOX), eprinomectin (EPR), and tunicamycin (TM)

Strain	MOX		EPR		TM	
	IC_50_ (nM) ± SD (nb of exp)	RF	IC_50_ (nM) ± SD (nb of exp)	RF	IC_50_ (µM) ± SD (nb of exp)	RF
Bristol N2	1.30 ± 0.06^c^ (4)	–	3.40 ± 0.37^b^ (4)	–	2.24 ± 0.09^b^ (4)	–
IVR10	2.83 ± 0.71 (4)	2.2	21.26 ± 4.65 (4)	6.3	6.05 ± 1.99 (7)	2.7
IVR10(Δ*pgp-9*)	1.06 ± 0.21^b^ (3)	0.8	2.74 ± 0.39^b^ (3)	0.8	2.76 ± 0.67^c^ (3)	1.2

N2B, wild-type Bristol N2; IVR10, IVM selected strain; IVR10(Δ*pgp-9*), IVR10 for which *pgp-9* was deleted; IC_50_, inhibition concentration 50%; RF, resistance factor, fold resistance relative to the N2B

^a^ *P* < 0.01; ^b^ *P* < 0.01; ^c^ *P* < 0.05 N2B or IVR10(Δ*pgp-9*) versus IVR10 (unpaired parametric *t*-test or unpaired *t*-test with Welch’s correction)

## Results

### *pgp-9* is required for IVR10 tolerance to other MLs and TM

**Fig. 1:**
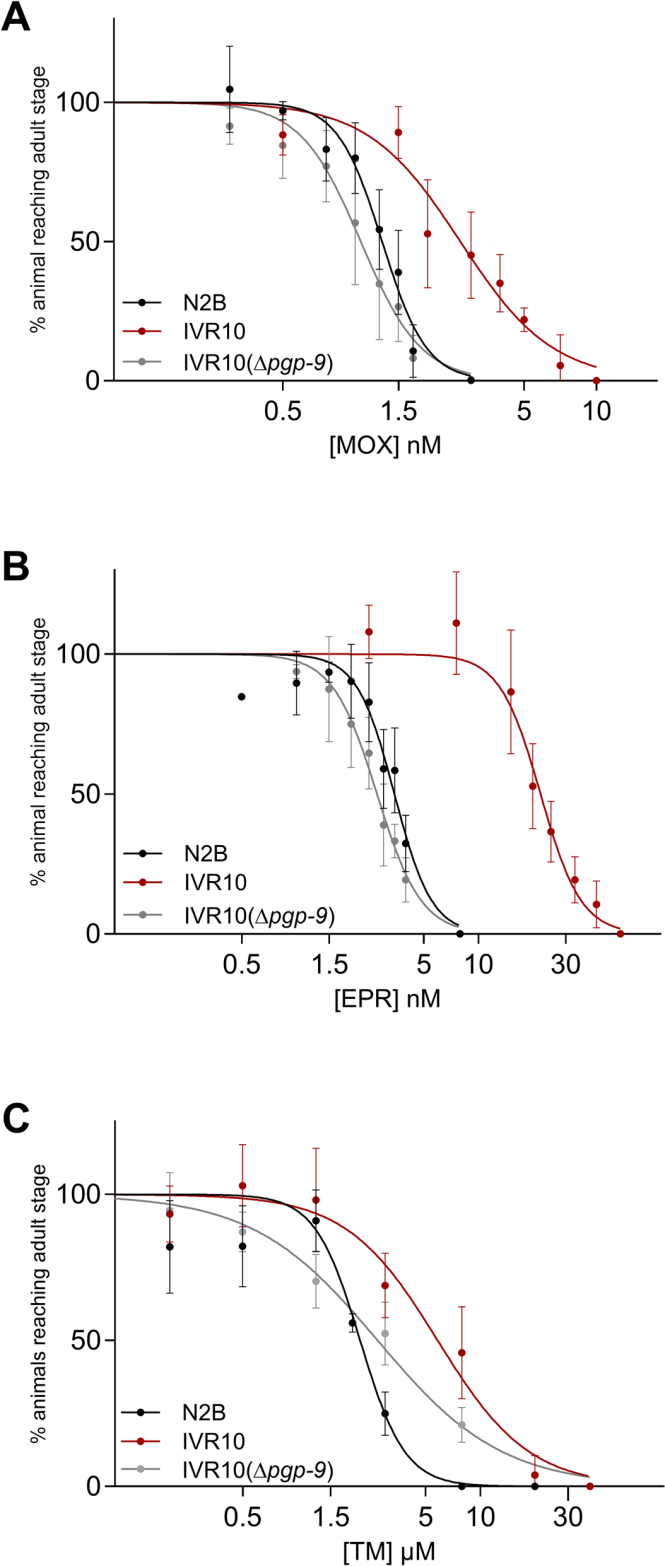
Effects of loss of *pgp-9* on cross-tolerance of IVR10. Sensitivities of IVR10(Δ*pgp-9*) to moxidectin (MOX) (**A**), eprinomectin (EPR) (**B**), and tunicamycin (TM) (**C**) on larval development assay (LDA). Dose–response curves to each compound (MOX, EPR, and TM) are compared with N2B and IVR10 as controls. Values represent the percentage of L1 reaching the young adult stage in the presence of increasing doses of the drug. Data are mean ± SD from 3–7 independent experiments. IC_50_ values for each strain are presented in Table 2

The IVR10 strain is a well-characterized *C. elegans* model of IVM resistance [12], previously shown to display cross-resistance to various AHs [14]. We previously showed that deletion of the ABC transporter gene *pgp-9* induced hypersensitivity to IVM in the IVR10 strain [23]. To further investigate the contribution of PGP-9 to drug detoxification, we first assessed whether it is required to maintain IVR10 cross-tolerance to MOX and EPR by characterizing the response of the *pgp-9* IVR10 knockout strain (IVR10(Δ*pgp-9*)) to these drugs. Sensitivities were assessed using larval development assays (LDAs), with IVR10 and N2B as resistant and susceptible reference strains. We confirmed our previous observations [14] that IVR10 was cross-resistant to MOX and EPR, as evidenced by significantly increased IC_50_ values and RFs of 2.2 and 6.3, respectively (Table 2; Fig. 1A, B). Surprisingly, *pgp-9* deletion in IVR10 led to complete reversal of the resistant phenotype of IVR10 to both MOX and EPR, characterized by a strong shift to the left of the dose–response curves and RFs below 1. Indeed, IVR10(Δ*pgp-9*) exhibited a significantly increased sensitivity to MOX, with a 2.6-fold lower IC_50_ value compared with IVR10 (1.06 ± 0.21 versus 2.83 ± 0.71 nM (*P* < 0.01)), and corresponding RFs of 0.8 versus 2.2 (Fig. 1A; Table 2). Similarly, sensitivity to EPR was also markedly increased, with a 7.8-fold lower IC_50_ value in IVR10(Δ*pgp-9*) compared with IVR10 (2.74 ± 0.39 versus 21.26 ± 4.65 nM (*P* < 0.01)), and RFs of 0.8 versus 6.3, respectively (Fig. 1B; Table 2). Importantly, the IC_50_ values of the IVR10(Δ*pgp-9*) strain for MOX and EPR were overall lower than those of N2B, although the differences were not statistically significant.

TM resistance has been reported in *C. elegans* mutants exhibiting amphid dye-filling defects [26]. Since IVR10 also displays this phenotype [14], we hypothesized that it might also be TM-resistant. We therefore tested TM efficacy in IVR10 and assessed whether it depends on PGP-9 by comparing sensitivities with the IVR10(∆*pgp-9*) mutant. IVR10 exhibited high tolerance to TM, as evidenced by a significant increase in the IC_50_ value compared with N2B: 6.05 ± 1.99 versus 2.24 ± 0.09 µM (*P* < 0.01), and an RF of 2.7 (Fig. 1C; Table 2). The deletion of *pgp-9* in IVR10 significantly reduced the tolerance to TM. This was supported by an RF of 1.2 and a 2.2-fold reduction in the IC_50_ value for the IVR10(Δ*pgp-9*) strain compared with IVR10: 2.76 ± 0.67 versus 6.05 ± 1.99 µM (*P* < 0.05) (Fig. 1C; Table 2).

These findings support that IVR10 displays cross-resistance to multiple MLs, as well as to TM, a structurally unrelated xenobiotic, with PGP-9 playing a central and indispensable role in the multidrug tolerance to these diverse compounds.

### IVR10(Δ*pgp-9*) is a Dyf mutant

**Fig. 2:**
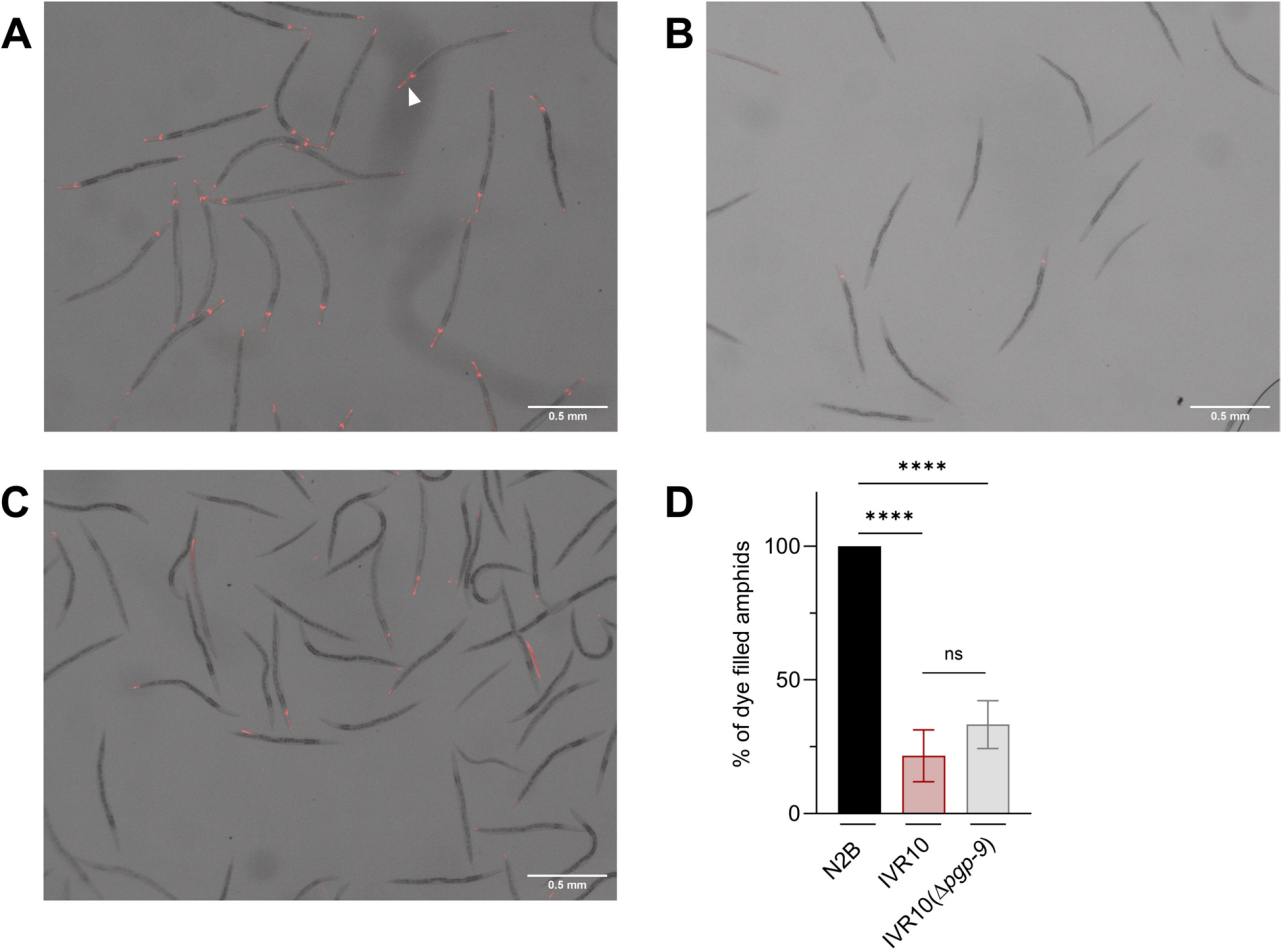
Dye-filling defect phenotype. Young adults N2B (**A**), IVR10 (**B**) and IVR10(Δ*pgp-9*) (**C**) were examined by fluorescence stereomicroscopy to visualize the dye filling of the amphids following staining with DiIC_12_(3) (DiI). The arrow indicates a set of amphids dyed red because of Dil absorption. **D** Percentage of dyed amphids, evaluated as the number of stained amphids normalized to the total number of worms treated. Data are expressed as mean ± SD from at least three independent experiments. ns *P* > 0.05; **** *P* < 0.0001 (one-way ANOVA followed by Tukey’s post hoc test)

The Dyf phenotype in nematodes reflects loss of the worm’s ability to internalize a fluorescent dye (Dil) that labels amphids, chemosensory neurons of the worm, and was previously associated with drug resistance [14, 24, 26, 28, 29]. Thus, we investigated whether the recovered drug sensitivity in IVR10(Δ*pgp-9*) was associated with a reversal of the Dyf phenotype by characterizing Dil uptake in the three strains: N2B, IVR10, and IVR10(Δ*pgp-9*). As shown in Fig. 2, while N2B worms displayed 100% of stained amphids, IVR10 exhibited the expected Dyf phenotype, with only 22% of worms stained (*P* < 0.0001 versus N2B). Surprisingly, IVR10(Δ*pgp-9*) maintained the Dyf phenotype of its parental strain IVR10, with 33% of worms stained with Dil (*P* < 0.0001 versus N2B, ns versus IVR10).

Our data demonstrate that, while *pgp-9* is essential for multidrug tolerance, the persistence of the Dyf in the IVR10 *pgp-9* knockout strain suggests that resistance strongly depends on other actors and cannot solely be attributed to amphid permeability changes. This observation then directed our attention toward transcriptional regulation by nuclear hormone receptors.

### Distinct yet complementary contributions of *pgp-9* and *nhr-8* in IVM tolerance

**Fig. 3:**
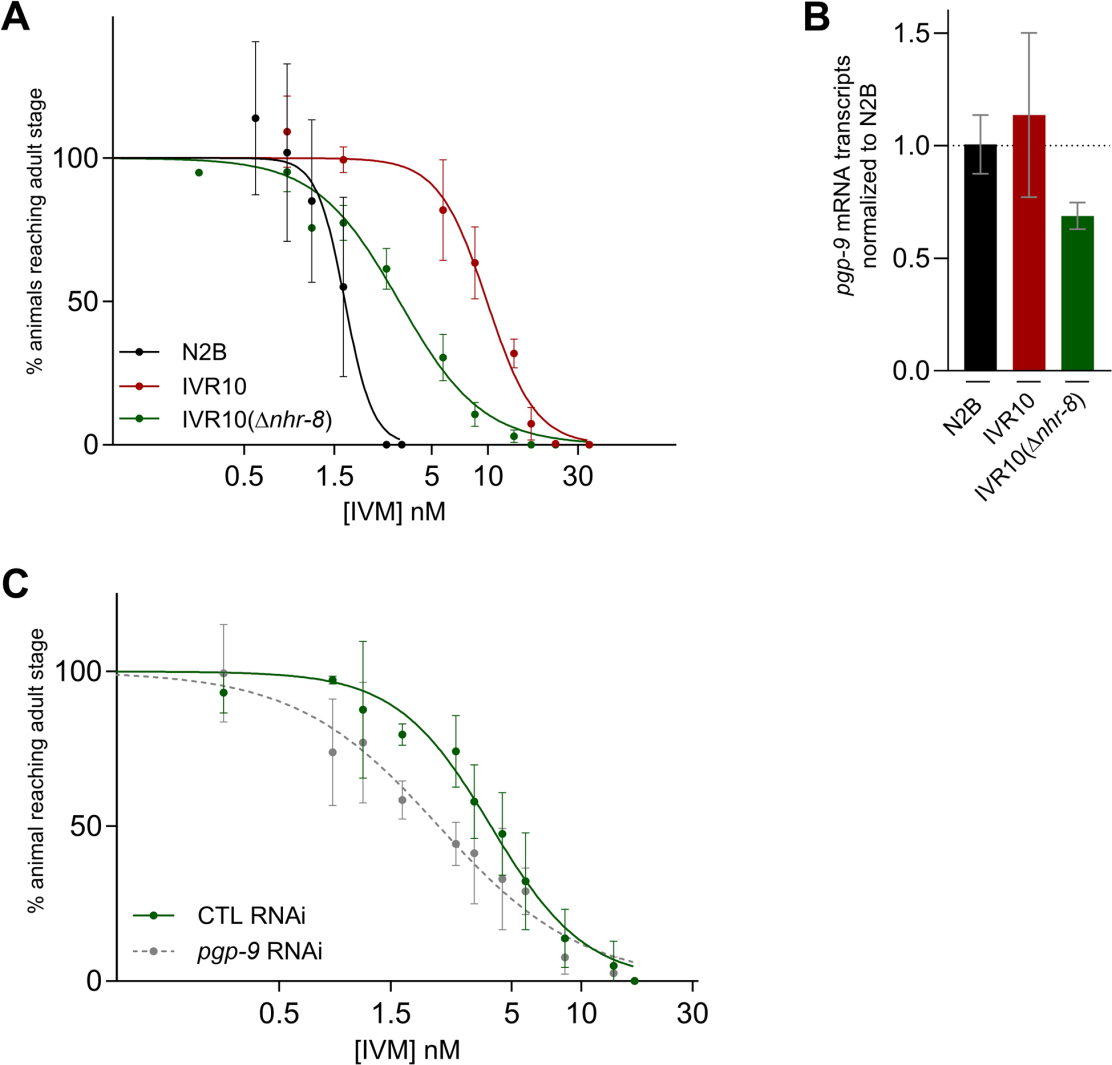
NHR-8 role in ivermectin tolerance and *pgp-9* regulation. **A** Effects of loss of *nhr-8* on tolerance of IVR10 to ivermectin (IVM) on larval development assay (LDA). Dose–response curves are compared with N2B and IVR10 as controls. For N2B and IVR10, values have already been published within a larger dataset [23]. **B** Quantification of *pgp-9* transcripts in N2B, IVR10, and IVR10(Δ*nhr-8*) by RT-qPCR. Data are expressed as fold change to the expression level of *pgp-9* in N2B and are normalized against the housekeeping gene *tba-1*. mRNA transcript levels are presented as mean ± SD from four independent experiments. mRNA levels were compared using a one-way ANOVA followed by Tukey’s post hoc multiple-comparisons test. **C** Additional effect of *pgp-9* silencing on sensitivity to IVM of IVR10(Δ*nhr-8*) on LDA. Worms were fed on HT115 bacteria transformed with either the L440 vector producing double-stranded RNA against *pgp-9* or an empty plasmid as a control. Dose–response curve of *pgp-9* silencing on IVR10(Δ*nhr-8*) is compared with IVR10(Δ*nhr-8*) fed on HT115-control. Values represent the percentage of L1 reaching the young adult stage in the presence of increasing doses of IVM. Data are mean ± SD from 3 or 4 independent experiments. IC_50_ values for each strain are presented in Table 3 (**A**) and Table 4 (**B**)

It was previously shown that NHR-8, a nuclear hormone receptor of *C. elegans*, mediates xenobiotic tolerance (e.g., IVM) in N2B by regulating the expression of detoxification factors, including ABC transporter genes such as *pgp-9* [11]. To clarify the role of NHR-8 in xenobiotic response and *pgp-9* regulation, we first studied its involvement in IVM tolerance in IVR10. To this end, we designed a knockout strain for *nhr-8* in the IVR10 background (IVR10 (Δ*nhr-8*)). The impact of *nhr-8* deletion on IVM sensitivity was compared with that of N2B and IVR10 using LDA. As expected, IVR10 showed a 6.2-fold higher tolerance than N2B. *nhr-8* loss-of-function led to a shift to the left of the IVM dose–response curve (Fig. 3A), supported by a 2.9-fold reduction in the IC_50_ value compared with that of IVR10 (9.75 ± 1.12 nM versus 3.35 ± 0.30, *P* < 0.001) (Table 3).

**Table 3:** Comparative sensitivities of *Caenorhabditis elegans* strains to ivermectin

Strain	IC_50_ (nM) ± SD (nb of exp)	RF
Bristol N2	1.57 ± 0.31^a^ (2^b^)	–
IVR10	9.75 ± 1.12 (3^b^)	6.2
IVR10(Δ*nhr-8*)	3.35 ± 0.30^a^ (3)	2.1

N2B, wild-type Bristol N2; IVR10, IVM selected strain; IVR10(Δ*nhr-8*), IVR10 for which *nhr-8* was deleted; IC_50_, inhibition concentration 50%; RF, resistance factor, fold resistance relative to the N2B

^a^ *P* < 0.001 N2B or IVR10(Δ*nhr-8*) versus IVR10 (one-way ANOVA followed by a post hoc Tukey’s test)

^b^ Values previously published within a larger dataset [23]

We then assessed the contribution of NHR-8 in regulating *pgp-9* expression by measuring *pgp-9* transcript levels in the IVR10(Δ*nhr-8*) strain (Fig. 3B). *Pgp-9* expression was unchanged in the IVR10 compared to N2B. Deletion of *nhr-8* in IVR10 caused a slight, though not statistically significant, decrease in *pgp-9* mRNA levels compared with both N2B and IVR10, indicating that NHR-8 does not play a prominent role in *pgp-9* expression in IVR10.

Because *pgp-9* was still expressed in IVR10(Δ*nhr-8*), we next examined whether silencing *pgp-9* in this strain could further reduce IVM tolerance. Gene silencing was achieved by RNAi specifically targeting *pgp-9* by feeding, with IVR10(Δ*nhr-8*) fed on bacteria (HT115) expressing an empty RNAi vector as a control. As expected, when fed on HT115 control, IVR10(Δ*nhr-8*) displayed a low sensitivity to IVM, as supported by an IC_50_ value of 4.17 ± 0.72 nM (Table 4; Fig. 3C). Importantly, silencing *pgp-9* further reduced IVM tolerance of the IVR10(Δ*nhr-8*) strain, as supported by a significant decrease in IC_50_ value from 4.17 ± 0.72 nM down to 2.44 ± 0.56 (*P* < 0.01) (Table 4; Fig. 3C).

**Table 4:** Combinatorial contributions of *pgp-9* and *nhr-8* to ivermectin sensitivity

Strain	RNAi^a^	IC_50_ (nM) ± SD (nb of exp)
IVR10(Δ*nhr-8*)	Control	4.17 ± 0.72 (4)
IVR10(Δ*nhr-8*)	*pgp-9*	2.44 ± 0.56^a^ (4)

*Pgp-9* was silenced in the IVR10(Δ*nhr-8*) strain

IVR10(Δ*nhr-8*), IVR10 for which *nhr-8* was deleted; IC_50_, inhibition concentration 50%

^a^ *P* < 0.01 IVR10(*nhr-8*) strain fed on *pgp-9* RNAi versus fed on control RNAi (unpaired parametric *t*-test)

Altogether, these data reinforce the importance of *pgp-9* in maintaining IVM tolerance and suggest that its protective role cannot be attributed to overexpression transcriptionally regulated by NHR-8, highlighting the complexity of xenobiotic resistance networks.

### *nhr-115*, *nhr-210*, and *nhr-226* do not participate in IVM tolerance

**Fig. 4:**
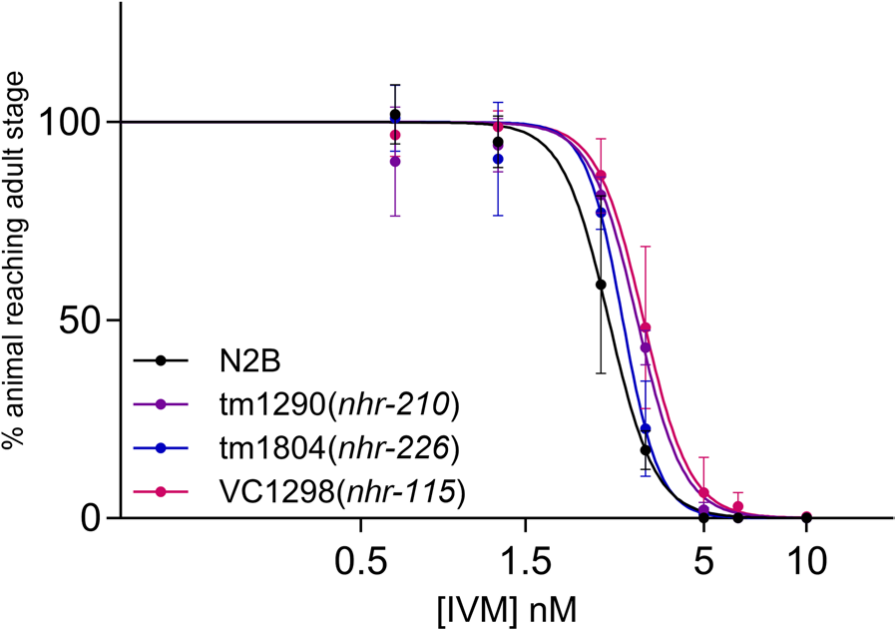
Contributions of NHR-210, NHR-226, and NHR-115 to ivermectin sensitivity. The effects of loss of *nhr-210*, *nhr-226*, and *nhr-115* in N2B on tolerance to ivermectin (IVM) were assessed on liquid larval development assay (LDA). Values represent the percentage of L1 reaching the young adult stage in the presence of increasing concentrations of IVM. Data are mean ± SD from 3 or 4 independent experiments. IC_50_ values for each strain are presented in Table 5

NHR-115, NHR-210, and NHR-226 were identified as potential regulators of *pgp-9* expression in previous studies [33, 34]. Thus, we explored their contribution to ML susceptibility in N2B, aiming to elucidate the broader transcriptional network regulating *pgp-9* and xenobiotic tolerance. We assessed the IVM tolerance of N2B strains individually lacking functional NHRs, i.e., NHR-115, NHR-210, and NHR-226, on liquid LDAs. Overall, individual loss of function of *nhr-210* and *nhr-226* did not significantly impact IVM sensitivity, as indicated by their respective superimposed dose–response curves (Fig. 4) and similar IC_50_ values (Table 5). Only the VC1298(*nhr-115*) strain exhibited a slightly increased IVM tolerance, as indicated by its significantly superior IC_50_ value when compared with N2B (Table 5). Taken together, these results support that the putative transcriptional regulators of *pgp-9* are not essential for ML tolerance.

**Table 5:** Impact of NHRs loss-of-function on sensitivities to ivermectin of *Caenorhabditis elegans* strains

Strain	Genotype	IC_50_ (nM) ± SD (nb of exp)	RF
N2B	Wild type	2.58 ± 0.42 (3)	–
tm1290	*nhr-210*	3.21 ± 0.07 (3)	1.2
tm1804	*nhr-226*	2.92 ± 0.13 (4)	1.3
VC1298	*nhr-115*	3.36 ± 0.37 (4)^a^	1.3

IC_50_, inhibition concentration 50%; RF, resistance factor, fold resistance relative to the N2B

^a^ *P* < 0.01 VC1298 versus N2B unpaired parametric *t*-test

## Discussion

This study deepens our understanding of drug resistance by exploring the functional role and regulation of one key PGP in IVM resistance, PGP-9 [23]. We relied on a relevant IVM-resistant strain, selected through stepwise exposure to the drug [12]. In this model, we previously showed that *pgp-9* deletion increased IVM sensitivity drastically [23]. Here, we further characterized the resistant profile of this strain by (i) demonstrating that the cross-resistance phenotype of IVR10 to other AHs, MOX and EPR, as well as to the endoplasmic reticulum stressor TM, is also dependent on *pgp-9*; (ii) evaluating the Dyf phenotype, typically associated to drug resistance [26, 31]; (iii) investigating the *pgp-9* regulatory mechanism by the nuclear hormone receptor NHR-8 [11] and transcriptional expression profile; and (iv) exploring the potential contribution of other NHRs to IVM tolerance.

As expected, the IVM-resistant strain IVR10 displayed cross-resistance to both MOX and EPR, with MOX displaying stronger efficacy than EPR. Our results are consistent with other findings in *C. elegans* [12, 14], suggesting MOX is a more potent drug against IVM-selected and MOX-selected drug-resistant worms compared with IVM and EPR. This is further supported by the higher efficacy exhibited by MOX compared with IVM against a *H. contortus* isolate resistant to both of these drugs [35], as well as MOX being more effective in reducing the burden of resistant and multiresistant isolates of *T. circumcincta* than IVM in lambs [36]. All together, these data suggest that the mechanisms underlying resistance to one ML share strong features with those conferring resistance to other MLs, while revealing variations in the potency of each compound. However, depending on the phenotypic assay used (LDA versus motility assay), differences in drug efficacy may also differ between strains, as evidenced by IVM having a higher potency than MOX or EPR against IVR10 and an *H. contortus* EPR-resistant isolate when considering worm motility [37].

We have previously shown that *pgp-9* deletion or knockdown in IVM-resistant *C. elegans* greatly increases IVM sensitivity [23]. Because IVR10 is cross-resistant to other AHs, we evaluated the sensitivity of IVR10(Δ*pgp-9*) to two additional MLs. Importantly, the cross-resistant phenotype of IVR10 to MOX and EPR was completely abolished owing to the deletion of *pgp-9*. These findings further prove the existence of common features in both tolerance and resistance mechanisms to AHs. In particular, PGP-9-mediated drug detoxification appears to be essential for IVR10 to tolerate and resist MLs. This represents the first in vivo evidence of recovered susceptibility to several MLs due to a loss of a PGP in nematodes. Future studies should also investigate whether PGP-9 contributes to the transport and detoxification of other AHs, such as monepantel, emodepside, and benzimidazoles, to further clarify its role in AH tolerance.

In N2B, mutations targeting genes involved in amphid structures, thus conferring the Dyf phenotype, are linked to TM resistance [26]. Because IVR10 is a Dyf strain [11], we examined its sensitivity to TM. We showed that IVR10 was resistant to this drug, confirming previous findings associating amphid defects with TM resistance [26]. The resistance of IVR10 to a wide range of structurally diverse drugs suggests that acquired resistance to IVM relies on overlapping and broad-spectrum mechanisms, including amphid defects. Interestingly, deletion of *pgp-9* completely reversed the resistant phenotype of IVR10 to TM. In this context, the impact of loss of *pgp-9* on cross-tolerance highlights the ability of PGPs to handle a wide range of substrates to protect cells from xenobiotics [3, 38, 39]. These elements indicate that PGP-9 has low substrate selectivity. Combined with its localization at major entry points such as the pharynx and intestine [23], this strongly supports a critical role for PGP-9 as an early defense mechanism against xenobiotic accumulation.

However, the regained sensitivities to both MLs and TM question the state of the Dyf phenotype in this IVR10 mutant.

Indeed, while the mechanistic advantage of displaying an amphid absorption defect and its associated mutation remains unknown, this phenotype has been repeatedly associated with ML resistance in both *C. elegans* [14, 24] and the parasitic nematode *H. contortus* [28, 29], and represents a major lead in the search and characterization of resistance-associated genes. However, amphid defective mutations do not necessarily confer drug resistance, as it was shown that the *che-6* Dyf mutant is susceptible to IVM [31]. Because IVR10 displays a Dyf phenotype and is multidrug-resistant [14], but *pgp-9* loss strongly alters its sensitivity to several compounds, we examined Dil absorption by amphids in IVR10(Δ*pgp-9*). Interestingly, this strain displayed an identical Dyf phenotype to the parental strain, IVR10, suggesting the conservation of resistance-associated genes and revealing intriguing dissociation between ML tolerance and Dyf phenotype. This suggests that amphid defects and PGP-9-mediated drug efflux may represent distinct, yet potentially complementary, mechanisms of drug tolerance or resistance. While PGP-9 plays a central role in conferring IVM tolerance in the IVR10 strain of *Caenorhabditis elegans*, its deletion strongly increases the sensitivity without restoring amphid dye uptake, indicating that the Dyf phenotype is not sufficient to maintain drug tolerance. Our observations support the hypothesis that amphid dysfunction, although associated with resistance, is probably part of a subset of tolerance actors and resistance genes. Loss of supporting actors, such as PGP-9, might endanger the resistant phenotype, resulting in hypersensitivity of resistant strains. These observations highlight the broad physiological adaptations acquired by IVR10 during the phase of selection for resistance.

Part of these actors could possibly be coordinated by transcriptional regulators such as NHR-8, which is known to modulate both xenobiotic responses and sensory functions in nematodes. Notably, NHR-8 regulates *pgp* genes in N2B, and *nhr-8* mutants display increased sensitivity to IVM [11]. Our results confirmed the critical role of NHR-8 in IVM tolerance, as the loss *of nhr-8* in IVR10 (IVR10(Δ*nhr-8*)) led to a significant increase in IVM sensitivity, supporting that NHR-8 modulates xenobiotic tolerance by regulating detoxification genes such as *cyp* or *pgp*, accordingly to a previous study [11].

Interestingly, analysis of *pgp-9* transcript levels revealed that its expression was not upregulated in the IVR10-resistant strain relative to the susceptible N2B strain. *Pgp-9* has been described as both upregulated [14, 18, 40–42] and downregulated [43, 44] in resistant or IVM-exposed nematodes, highlighting that its contribution to resistance cannot be attributed solely to its overexpression.

*Pgp-9* deletion of *nhr-8* resulted in only a slight, nonsignificant decrease in *pgp-9* mRNA. Although these observations contrast with previous findings [11], they align with another study in which *nhr-8* loss-of-function in N2B did not impact *pgp-9* expression levels [26]. Taken together, these findings support the diverse and context-dependent regulation of detoxification genes, including those controlled by NHR-8.

Because NHR-8 does not drive *pgp-9* transcriptional overexpression in our context, we evaluated whether it could have an additive effect on IVM tolerance by silencing *pgp-9* in the IVR10(Δ*nhr-8*) mutant. Interestingly, *pgp-9* knockdown further increases IVM efficacy, confirming that *pgp-9* contributes to tolerance independently of its transcriptional regulation mediated by NHR-8.

Together, these data imply that the role of *pgp-9* in IVM tolerance may also be regulated at post-transcriptional or post-translational levels, possibly involving modulation of its transporter activity or stability. Such mechanisms align with the complex and multilayered regulatory networks controlling xenobiotic responses, where transcriptional changes represent only one facet. While these findings clarify that increased *pgp-9* transcription is not the driving force behind its contribution to tolerance in IVR10, additional regulatory layers or involvement of other *pgp* family members cannot be excluded. The intricacies of ABC transporter regulation and their interplay in xenobiotic tolerance warrant further investigation to fully elucidate the tolerance and resistance mechanisms at play.

Because PGP-9 displays a central role in IVM tolerance, we investigated whether other putative transcriptional regulators might impact drug susceptibility in N2B. A previous study assembled cliques based on large-scale RNAi experiments, i.e., groups of genes that are highly coexpressed with each other across these RNAi experiments [33]. Among these cliques, *pgp-9* was coexpressed with *nhr-210*, *nhr-226*, and *nhr-115*, along with *cyp* and *ugt* genes. Thus, we postulated that these NHRs might influence *pgp-9* expression and the tolerance to IVM. None of the N2B *nhrs* loss-of-function strains exhibited significant altered sensitivity to the drug, except for *nhr-115*, which is predicted to be a transcriptional regulator involved in innate immune response pathways [45]. Although the loss of NHR-115 increased tolerance to IVM, the extent of this effect may be insufficient to consider NHR-115 as a suitable target to improve treatments. Surprisingly, we expected that loss of function of *nhr-210* would have impacted sensitivity to IVM, as *pgp-9* was predicted to be one of its targets in a *C. elegans* Parkinson’s disease model [34]. These results further highlight the complexity of the regulation of detoxification genes. It is also plausible that several NHRs exert transcriptional control over the same gene [46], and that NHRs involved may be dependent on the context, including the xenobiotic at play. For instance, two previous studies suggest that NHR-8 contributes to xenobiotic tolerance (including TM) partly through *pgp-3* regulation [11, 26], whereas another one reported that NHR-8 and *pgp-3* exhibit common but different spectra of toxin responses [47]. This notably illustrates that the NHR control over *pgp* may not be restricted to a single regulatory pathway. Assessing the effects of combinatorial *nhr* knockouts on *pgp-9* expression and drug sensitivity would provide valuable insights into the mechanisms of gene regulation by NHRs.

## Conclusions

Our study further demonstrates that PGP-9 holds a key role in tolerance not only to IVM but also to related AHs from the ML class, as well as other xenobiotics such as TM. Although deletion of *pgp-9* significantly reduced drug tolerance, the IVR10 strain retained key ML-resistance-associated genes, as illustrated by the Dyf phenotype in IVR10(Δ*pgp-9*). This suggests that, while *pgp-9* may not be directly a gene of ML resistance, its inhibition could nonetheless be leveraged to counter drug resistance. PGP-9 appears as a key player in drug tolerance and is likely required for the acquisition of resistance genes, and may remain active to help maintain resistance once established. We further deepened our understanding of the mode of action of PGP-9 by showing that its protective effect against drug toxicity in IVR10 could not be associated with overexpression, nor transcriptional regulation by NHR-8. Instead, our results suggest an additive effect in restoring sensitivity in IVR10 when targeting NHR-8 and PGP-9, underscoring the importance of integrating both transporter function and regulatory pathways to fully understand nematode xenobiotic resistance. In particular, PGP-9 deserves continued investigation, as it appears to have a distinct and specific role in ML tolerance compared with its paralogs in *C. elegans*. Future studies should focus on elucidating the post-transcriptional and post-translational regulation of PGP-9. Investigating other members of the PGP family in *C. elegans* will also be essential to reveal potential compensatory mechanisms and substrate specificity. Additionally, exploring the molecular basis of the persistent Dyf could uncover neuronal or structural changes contributing to resistance. Together, these approaches will advance our understanding of the complex interplay between transporter activity, gene regulation, and physiological adaptations that confer resistance to MLs. Such insights are critical to overcome drug resistance in parasitic nematodes, with significant implications for controlling helminth infections in human and veterinary medicine.

## Data Availability

The datasets generated and analyzed during the current study are available at: 10.57745/MGNYAL.

## Abbreviations

ABC: ATP-binding cassette
AH: Anthelmintic
CGC: Caenorhabditis Genetics Center
Dyf: Dye-filling defect
EPR: Eprinomectin
GluCl: Glutamate-gated chloride channel
IC_50_: Inhibitory concentration 50%
IVM: Ivermectin
IVR10: Ivermectin-resistant *C. elegans* to 10 ng/ml of ivermectin
LDA: Larval development assay
ML: Macrocyclic lactone
MOX: Moxidectin
mRNA: Messenger RNA
N2B: N2 Bristol
NGM: Nematode growth medium
NHR: Nuclear hormone receptor
PGP: P-glycoprotein
RF: Resistance factor
RNAi: Interfering RNA
RT-qPCR: Reverse-transcription quantitative polymerase chain reaction
TM: Tunicamycin
